# Niobium-Decorated Multiwalled Carbon Nanotubes for Voltammetric Detection of Paracetamol

**DOI:** 10.1021/acsomega.4c10722

**Published:** 2025-05-07

**Authors:** Rafael H. de Oliveira, Monize M. da Silva, Claudio T. de Carvalho, Daniel A. Gonçalves, Diogo D. dos Reis

**Affiliations:** † Institute of Physics, 186079 Federal University of Mato Grosso do Sul—UFMS, Campo Grande 79070-900, Mato Grosso do Sul, Brazil; ‡ Faculty of Exact Sciences and Technology, Federal University of Grande Dourados—UFGD, Dourados 79804-970, Mato Grosso do Sul, Brazil; § Institute of Exact Sciences—ICE, Federal University of Amazonas—UFAM, Manaus 69080-900, Amazonas, Brazil

## Abstract

This study investigates the synthesis and characterization of a nanostructured sensor based on multiwalled carbon nanotubes (MWCNTs) decorated with niobium oxide (Nb_2_O_5_) nanoparticles. The MWCNT-Nb_2_O_5_ composite was synthesized using a chemical method that ensured a homogeneous distribution of Nb_2_O_5_ on the MWCNT surface. These nanocomposites were used as modifiers of glassy carbon electrodes for the voltammetric detection of paracetamol in aqueous media. The structural and compositional properties of the material were comprehensively characterized using scanning electron microscopy (SEM), energy-dispersive X-ray spectroscopy (EDS), X-ray diffraction (XRD), X-ray photoelectron spectroscopy (XPS), and thermal analysis (TGA-DSC/DTG). Electrochemical analyses, including cyclic voltammetry and square wave voltammetry in Britton–Robinson buffer solution, were employed to assess the performance of the GCE/MWCNT/Nb sensor. The sensor exhibited high sensitivity and precision for paracetamol detection, maintaining reliable performance even in the presence of interferents such as epinephrine and tryptophan. With detection and quantification limits of 0.100 and 5.00 × 10^–10^ mol L^–1^, respectively, and a recovery rate of 103%, the sensor outperformed several existing electrochemical methods. These findings underscore the suitability of the MWCNT/Nb nanocomposite as a robust platform for precise and accurate paracetamol detection.

## Graphical abstract

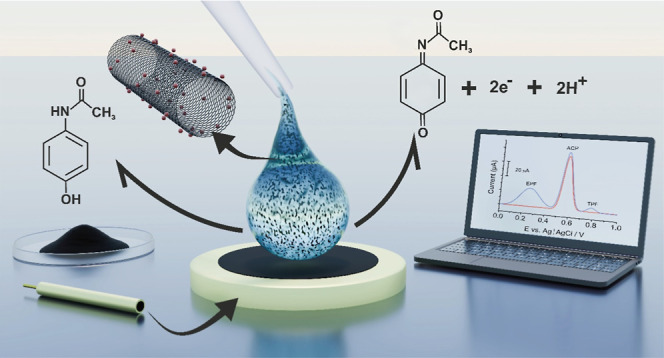

## 1 Introduction

Growing concerns about emerging water contaminants have spurred the development of more sensitive and selective detection methods. Such pollutants, including pharmaceuticals, personal care products, and industrial chemicals, often evade conventional water treatment systems. The detection of these contaminants is critical for identifying potential health and environmental hazards, guiding the development of effective mitigation and treatment strategies.^1–4^

While several detection techniques, such as chromatographic and spectroscopic methods, are available, electroanalytical approaches offer unique advantages due to their sensitivity and specificity, simplicity, and potential for real-time, in situ monitoring.^5–7^ Compared to chromatographic and spectroscopic methods, they require minimal sample preparation and are more cost-effective. Additionally, electrochemical sensors offer greater portability and shorter analysis times, making them especially suitable for continuous environmental monitoring in both laboratory and field settings.^1,8,9^

Electrochemical sensors have revolutionized analytical protocols, particularly in the detection of emerging contaminants relevant to public health. The incorporation of metal oxide nanomaterials (MONs) into sensor platforms offers new possibilities to further enhance the sensitivity and selectivity of these detection systems.^10–12^

Several MONs, including copper­(II) oxide (CuO),^13^ tin­(II) oxide (SnO),^14^ zinc oxide (ZnO),^15^ nickel oxide (NiO),^4^ indium oxide (In_2_O_3_),^16^ titanium oxide (TiO_2_),^17^ and tungsten oxide (WO_3_),^18^ have been widely explored for sensing applications due to their high sensitivity, fast response times, and stability, as well as cost-effectiveness, thanks to their straightforward fabrication processes. Among these, niobium-based oxides have emerged as particularly promising due to their catalytic properties and compatibility with carbon nanomaterials.^19–21^

Niobium-based metal oxide nanomaterials have garnered significant attention due to being highly suitable for applications in electrical and electronic devices, catalysis, and gas sensors.^19,22–24^ The versatile functionality and promising performance of niobium in these fields highlight its potential for widespread industrial use and technological advancement. These oxides are mainly constituted by octahedral structures (i.e., NbO_6_) but present structural variations (NbO, Nb_2_O_5_, etc.) that influence their efficiency in their acid catalysts. Amorphous niobium pentoxide, containing both Brønsted and Lewis acid sites, is effective in water media, which is crucial for sensor applications.^25^ MON-based niobium also plays a key role in achieving good gas detection performance in conventional resistance-type gas sensors. Thus, reducing the size of NbO nanoparticles enhances the surface-to-volume ratio, and integrating them with conductive carbon nanomaterials effectively addresses this limitation.^26^

Despite the limited surface area and suboptimal electrochemical performance of pure Nb_2_O_5_, its functionality can be enhanced by integrating support media such as reduced graphene oxide (rGO), molecularly imprinted polymers (MIP), or carbon nanotubes. The synergistic interaction between niobium oxides and these materials enhances stability and increases the electroactive surface area. Due to its stable phase, nontoxic nature, and promising electrocatalytic properties, niobium oxide is increasingly being explored for sensor applications. However, studies on its performance in electrochemical sensors remain limited, providing an opportunity for further investigation.^27^

Conversely, numerous studies highlight the potential of niobium oxide-based nanocomposites in sensor technologies. For instance, core–shell niobium (V) oxide@molecularly imprinted polythiophene nanoreceptors have been effectively employed for real-time creatinine analysis, achieving a remarkable detection limit of 34.0 × 10^–12^ mol L^–1^ with high selectivity in human saliva.^28^ However, these systems face challenges related to complex synthesis and nonspecific retention of similar compounds. Additionally, a ternary nanocomposite of reduced graphene oxide (rGO) and niobium oxide–niobium carbide has shown a low detection limit (1.60 × 10^–9^ mol L^–1^) for the anticancer drug methotrexate, further illustrating the potential of niobium-based materials in electrochemical sensors.^27^

Both MWCNTs and rGO materials are exceptional materials for electrochemical sensors. However, MWCNTs offer advantages over rGO including higher electrical conductivity, superior mechanical strength, flexibility, and a cylindrical structure that enhances surface area and porosity for improved adsorption and sensitivity.^29^ MWCNTs also exhibit robust chemical and thermal stability, ensuring longevity and ease of functionalization for enhanced selectivity. Unlike rGO, MWCNTs are less prone to aggregation, maintaining an effective surface area over time.^30^ Incorporating niobium into carbon nanotubes further enhances sensor catalytic activity and active surface area, facilitating selective detection of contaminants in complex aqueous environments.^31^

This research explores the synthesis of niobium-decorated MWCNTs for use as MON-based sensors, aimed at modifying glassy carbon electrodes to enhance the voltammetric detection of emerging aqueous contaminants. Various characterization techniques will be applied to confirm the structural and compositional integrity of the MON-based sensors. This approach aims to achieve a uniform distribution of niobium particles on the carbon nanotubes, maximizing sensor efficiency. The electroactive behavior of the GCE/MWCNT/Nb system will be investigated using cyclic voltammetry and square wave voltammetry in buffer solutions. As a proof of concept, paracetamol will be used as an analyte, with epinephrine and tryptophan as potential interferents, demonstrating the high sensitivity and accuracy of the proposed sensor platform for detecting emerging contaminants.

## 2 Experimental Section

### 2.1 Materials

The materials used in this investigation included titanium isopropoxide (TiP, 97%) and hydrochloric acid (HCl, 36%) from Sigma-Aldrich; sodium hydroxide (NaOH) and nitric acid (HNO_3_, 65%) from Dinâmica Química Contemporânea Ltd.a.; sulfuric acid (H_2_SO_4_, 98%) from Neon Comercial Reagentes Analíticos Ltd.a.; and Niobium Ammonium Oxalate (OAN) (NH_4_[NbO­(C_2_O_4_)­2­(H_2_O)]·(H_2_O)*n*) from CBMM (Companhia Brasileira de Metallurgia e Mineração). Hydrogen peroxide (H_2_O_2_, 30 Vol 9%) from Exôdo Científica, absolute ethyl alcohol (99.5%) from CRQ Produtos Químicos, isopropanol from Sciavicco Comércio e Indústria Ltd.a., and ammonium hydroxide (NH_4_OH, 28%) from Cromaline Química Fina were also employed. Multiwalled carbon nanotubes (MWCNTs) with >93% purity were provided by the Nanomaterials Laboratory at UFMG. Detailed characterization of the MWCNTs is available in the Supporting Information (Figures S1 and S2).

### 2.2 Synthesis of the MWCNT/Nb Nanocomposite

Figure 1 schematically illustrates the synthesis of the MWCNT/Nb nanocomposite. The synthesis began with the surface modification of the multiwalled carbon nanotubes (MWCNTs) to introduce oxygenated functional groups. A solution of HNO_3_ and H_2_SO_4_ in a 1:3 (v/v) ratio was prepared to a final volume of 30 mL, into which 500.0 mg of MWCNTs were added. The mixture underwent to ultrasonic treatment for 2 h at room temperature, followed by a resting period of 15 h.

**Figure 1:**
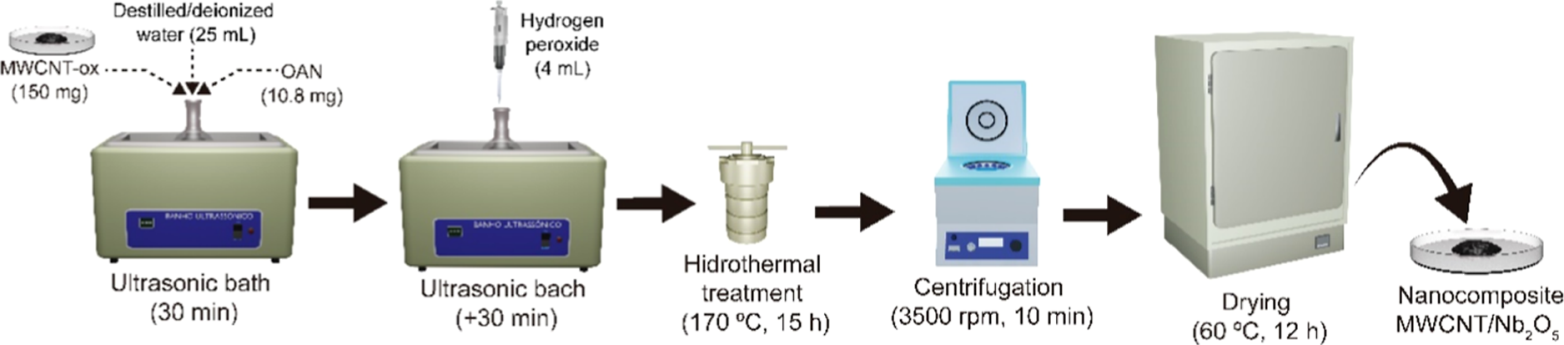
Illustrative scheme of preparation of the MWCNT/Nb nanocomposite and hydrothermal treatment.

After this, HCl was added to the solution (17% of the total liquid volume), and the mixture was allowed to react for 30 min before being neutralized with NH_4_OH. The solution was filtered under vacuum using quantitative filter paper (grade 41), and the solid obtained was washed with deionized water until the pH reached 5.5. The oxidized MWCNTs, named here by MWCNT-oxi, were then dried at 60 °C for 12 h.

The functionalization of MWCNT-oxi with niobium pentoxide (Nb_2_O_5_) nanoparticles was performed using the peroxide oxidation method (OPM), followed by hydrothermal treatment.^32,33^

For the functionalization, 10.8 mg of Niobium Ammonium Oxalate (OAN) was mixed with 150 mg of MWCNT-oxi in 25.0 mL of deionized water and subjected to ultrasonic treatment for 30 min. Then, 4.00 mL of hydrogen peroxide (30% v/v) was added to form a colloidal solution of niobium peroxo-complex (NPC). The mixture was kept in the ultrasonic bath for an additional 30 min^34^ During this process, OAN is converted into Nb_2_O_5_ and ammonium oxalate ((C_2_O_4_(NH_4_)_2_), as shown in generic eq 1.

1 $${2NH}_{4}\left[NbO{\left(C_{2}O_{4}\right)}_{2}{\left(H_{2}O\right)}_{2}\right]{\cdot nH}_{2}O+\left(H_{2}O+H_{2}O_{2}\right)n\to {Nb}_{2}O_{5}+4\left(C_{2}O_{4}{\left({NH}_{4}\right)}_{2}\right)$$

The resulting mixture was transferred to a 35 mL Teflon container and placed in a stainless-steel autoclave, where it underwent hydrothermal treatment at 175 °C for 15 h. The material was then washed twice with deionized water, centrifuged at 3500 rpm for 10 min, and dried at 60 °C for 12 h, yielding the MWCNT/Nb nanocomposite.

### 2.3 Characterization of the MWCNT/Nb Nanocomposite

The material was characterized using simultaneous TG-DSC/DTG analysis with a NETZSCH STA 449F3 Jupiter thermobalance. The experiment was carried out at a heating rate of 10 °C/min in an air atmosphere with a flow rate of 50 mL/min and a sample mass of approximately 3 mg. The heating ranges between 30 to 1000 °C. Scanning electron microscopy (SEM) coupled with energy-dispersive X-ray spectroscopy (EDS) was performed using a JEOL JSM-6380LV microscope and a Thermo Scientific Noran System SIX EDS detector, operating at an electron beam acceleration voltage of 20 kV. Samples were prepared by suspending them in isopropanol, applying them onto glass substrates, and coating them with a thin layer of gold via cathodic sputtering. X-ray diffraction (XRD) was employed to identify crystalline phases, using a D/teX Ultra 250 detector with a Cu–Kα radiation source (λ = 1.54186 Å). The X-ray generator was operated at 40 kV and 20 mA. The measurement was conducted in 1D scan mode over a range of 4.0000–70.0000°, with a scan step of 0.0200° and a scan speed of 2.0000°/min. The X-ray photoelectron spectroscopy (XPS) analyses were conducted using an Escalab Xi + instrument (Thermo Scientific). The incident monochromatic X-ray beam, generated from an aluminum (Al) target at 1486.6 eV, was focused on a 650 μm diameter spot surface. To acquire high-resolution spectra of the Ni peaks, the electron energy analyzer was operated with a pass energy of 20 eV. A step size of 0.1 eV was employed, and each peak was scanned 30 times in three positions of the sample. Prior to transferring the samples to the analysis chamber, the introduction chamber underwent evacuation for 12 h reaching a final pressure of 4 × 10^–8^ mbar. Data collection occurred under a base pressure of 8 × 10^–10^ mbar in the analysis chamber. The binding energies of the photoelectrons were calibrated using the C (1s) photoelectron line (BE = 284.8 eV). These methods provide a comprehensive understanding of the structural, morphological, chemical and thermal properties of nanocomposites.

### 2.4 Electrochemical Measurements

The modified electrode was fabricated by depositing a composite solution onto the glassy carbon electrode (GCE). Before applying the solution, the electrode was mechanically polished with an alumina dispersion for 5 min. For the addition of the solution of MWCNT decorated with niobium nanoparticles (i.e., MWCNT/Nb at a concentration of 1.00 mg/mL in ultrapure water), an automatic micropipette (Eppendorf, model Multipette E3X) was used. A 10.00 μL aliquot of the solution was added onto the GCE and subsequently immobilized with a 5.00% (w/v) of 10.00 μL Nafion-methanol mixture. This resulted in the modified electrodes, GCE/MWCNT/Nb, which were then used for electrochemical measurements. The same preparation procedure used for GCE/MWCNT/Nb was applied to the comparative electrode GCE/MWCNT/Ti as a second material for comparative tests and to evaluate the performance of the proposed sensor. Further information on the synthesis, characterization, and preparation of GCE/MWCNT/Ti can be found in previous literature published by de Oliveira et al. 2023.^2^

Standard solutions were prepared using stock solutions of epinephrine, paracetamol, and tryptophan, all with a concentration of 0.0100 mol L^–1^, in ultrapure water (resistivity ≥18.2 mol L^–1^ Ω cm, Dionex IC Pure water purification system, Thermo Scientific). These solutions were used to fortify the samples during the addition and recovery experiments, conducted by cyclic voltammetry (CV) or square wave voltammetry (SWV). All experiments were conducted in a 10.0 mL glass electrochemical cell with a three-electrode system, under constant magnetic stirring. After each triplicate, a 30 s agitation was performed to homogenize the solutions and remove electrogenerated products from the electrode surface.^35^

A glassy carbon electrode with a diameter of 3.00 mm was used as the working electrode (W.E.), a platinum plate served as the auxiliary electrode (A.E.), and an Ag|AgCl reference electrode (in saturated KCl) was used as the reference (R.E.). Analyte and interferent samples were prepared in tap water filtered through a 25 μm cartridge, adjusted to a concentration of 1.00 mmol L^–1^ and a pH of 7.0, and stored in glass containers for addition and recovery experiments. Britton–Robinson buffer (B–R) was chosen to maintain the pH at 7.0. The inverse axis method and multienergy calibration (MEC) were evaluated to generate calibration curves due to the robustness they exhibited, especially in situations where a complex sample matrix requires the application of the standard addition method.^35–37^

Square wave voltammetry (SWV) and cyclic voltammetry (CV) measurements were performed using a μ-Autolab Type III potentiostat/galvanostat (Metrohm Autolab, Utrecht, Netherlands) controlled by Nova 2.1 software. The measurement parameters were adjusted, as follows: step potential/mV: 5.0; modulation amplitude/mV: 50.0; time interval/s: 0.20; and Scan rate (ν)/(V s)-1:0.025. For SW voltammograms, raw data were preprocessed by baseline correction, followed by peak deconvolution using Origin software (version 8.5).

## 3 Results and Discussion

### 3.1 Morphological and Structural Characterization of the MWCNT/Nb Nanocomposite

Simultaneous TGA-DSC/DTG analyses (Figure 2) were performed to evaluate the thermal behavior of pristine MWCNT (Figure 2A) and the MWCNT/Nb nanocomposite (Figure 2B). The two samples, pristine MWCNT and MWCNT/Nb, were prepared by treatment with a concentrated mixture of nitric and sulfuric acids. According to Figure 2A, the MWCNT shows a slight mass loss of 4.5% (TGA) above 350 °C, stabilizing at approximately 440 °C. This mass loss is likely due to the presence of oxidizing functional groups covalently bound to the surface of the MWCNT, which favors decomposition at a lower temperature.

**Figure 2:**
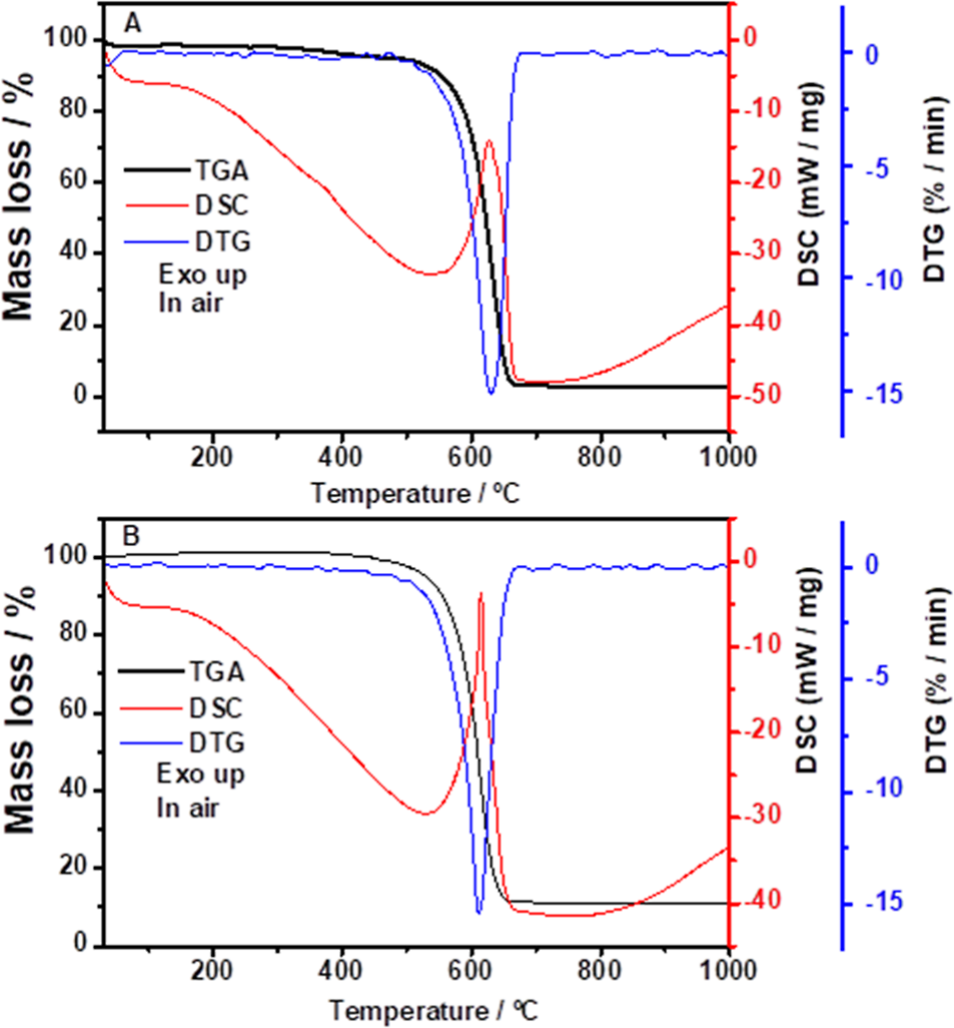
(A,B) TGA-DSC/DTG curves in an air atmosphere for the pristine MWCNT (A) and MWCNT/Nb (B) samples.

Thermal stability is maintained up to 510 °C, after which the decomposition of the carbon skeleton begins. This decomposition is completed at 678 °C, accompanied by an exothermic peak observed in the DSC curve at 627 °C and a DTG peak at 630 °C. On the other hand, the niobium-doped material, MWCNT/Nb, starts to decompose gradually at 450 °C and continues until complete decomposition at 659 °C. This process is characterized by an exothermic peak at 614 °C and a DTG peak at 611 °C. For the decomposition of the MWCNT and MWCNT/Nb, it is possible to observe the formation of stable residues of 3.0% and 11.4%, respectively. The 11.4% residue is likely associated with the formation of Nb_2_O_5_, originating from the niobium content incorporated onto the surface of the MWCNT.

The morphological and structural characterization of the MWCNT/Nb nanocomposite plays a pivotal role in understanding its superior electrochemical performance. The SEM images (Figure 3) revealed a well-dispersed network of MWCNTs modified with niobium oxalate (MWCNT/Nb) with no significant agglomeration, ensuring a high surface area for interaction with analytes. This morphology facilitates the effective adsorption and subsequent detection of target molecules, such as paracetamol, even in the presence of interferents. Particles attributable to Nb precursor precipitation or niobium oxide formation are not distinctly observed.

**Figure 3:**
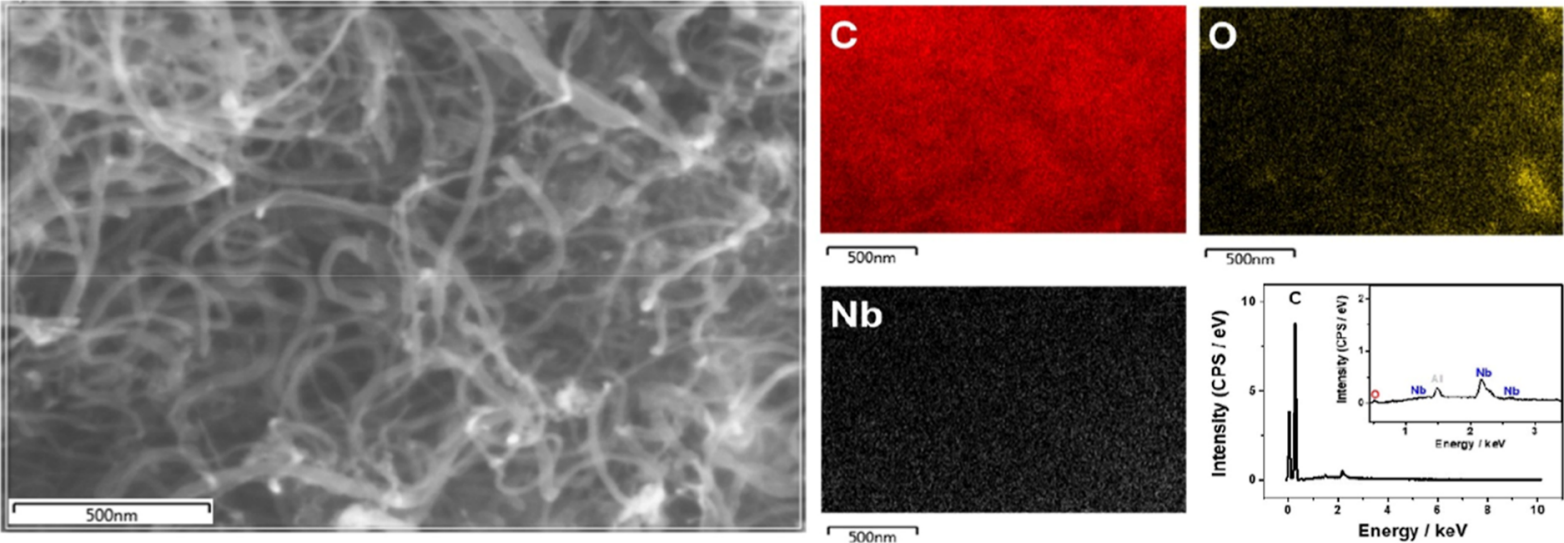
SEM image of oxidized MWCNT modified with niobium oxalate (MWCNT/Nb) on the left. Carbon (C), oxygen (O), and niobium (Nb) mapping and EDS for MWCNT/Nb on the right.

EDS mapping (Figure 3) confirms the presence of niobium, suggesting the formation of niobium complexes as isolated sites on the oxidized MWCNTs. It also provides critical evidence of the uniform distribution of niobium across the surface of the MWCNTs. This uniformity is crucial, as it ensures consistent catalytic activity throughout the electrode surface, reducing localized deficiencies in electrochemical response. The EDS spectrum further verifies the elemental composition, showing a uniform distribution with peaks for niobium (2.2 and 2.6 keV), oxygen (0.5 keV), and carbon (0.3 keV). A peak for aluminum (1.6 keV) is also detected, arising from the catalyst used in the synthesis of MWCNTs. Additionally, EDX analysis was employed to quantitatively characterize the elemental composition of the oxidized MWCNTs modified with niobium oxalate (MWCNT/Nb). This analysis confirms that carbon (98.8 wt %), oxygen (0.5 wt %), and niobium (0.60 wt %) are the predominant elements in the MWCNT/Nb composite and negligible percentages of other elements (<0.10 wt %).

XRD diffractogram exhibits distinct low-intensity diffraction peaks at 2θ = 32.6 and 47.6° (Figure 4a), which can be indexed to a pseudohexagonal crystallographic structure (TT-Nb_2_O_5_), assigned to crystallographic planes (110) and (002) (JCPDS card no. 28–0317). The weak-intensity bands observed are attributed to the low synthesis temperature, which may have affected the material’s crystallinity. Graphitic structure diffraction peaks are observed at 2θ = 26.1, 43.2, and 53.4° (JCPDS card no. 41–1487), corresponding to crystallographic planes (002), (100), and (004), respectively. This result indicates that MWCNTs are well graphitized. Additionally, weak peaks at 2θ = 37.2 and 45.3°, assigned to aluminum crystallographic planes (111) and (200), respectively (JCPDS card no. 04–0787), originated from the catalyst template in the MWCNTs. The survey XPS spectrum exhibits peaks of C (1s), O (1s) and Nb (3d), as shown in Figure 4b. The high-resolution XPS spectrum of the Niobium 3d peaks (Figure 4b inset) reveals only two peaks at 208.5 and 211.3 eV, which are characteristic of the Nb_2_O_5_ compound. This observation demonstrates that niobium is exclusively oxidized in the Nb^5+^ state.

**Figure 4:**
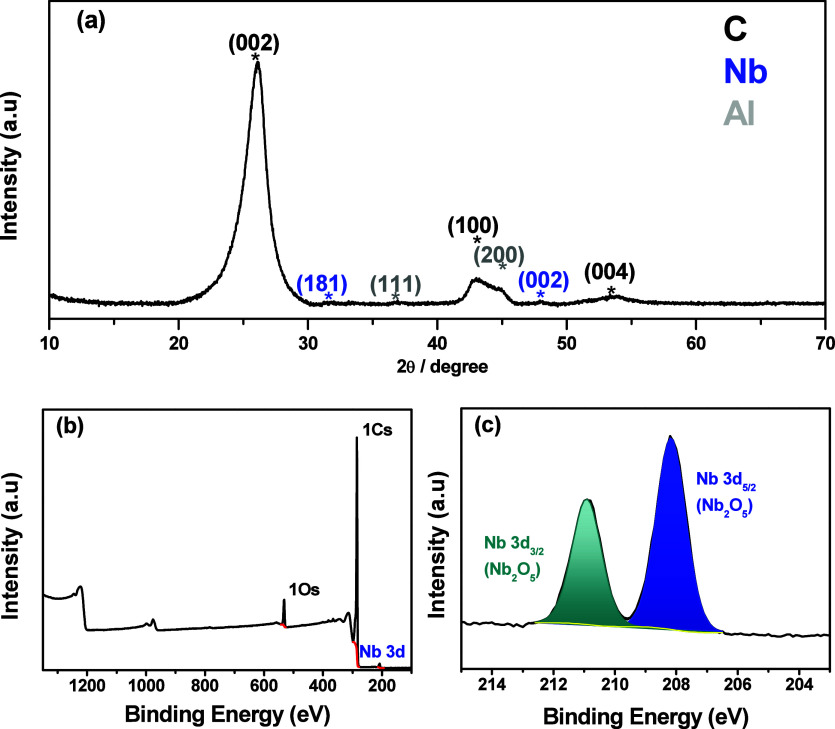
XRD diffractogram (a), XPS survey spectrum (b), and high-resolution XPS spectrum of the Nb 3d peaks (c).

### 3.2 Electrochemical Characterization

Figure 5 ilustartes the electrochemical behavior of GCE and GCE/MWCNT/Nb electrodes in a 0.50 mol L^–1^ H_2_SO_4_ solution, emphasizing the significance of this study. Cyclic voltammetry, conducted within a potential range of −0.50 to 1.5 V at a scan rate of 25 mV s^–1^, offered valuable insights. Analysis of the CGE electrode revealed its electrochemical inactivity under the specified experimental conditions, as documented by Benck, et al. No redox pair was observed in the corresponding cyclic voltammogram for the GCE electrode.^38^ The graphical representation of this phenomenon in Figure 5, illustrates a narrow and nearly horizontal line, indicating the absence of electrochemical reactions on the surface of the GCE electrode under these circumstances. However, introducing carbon nanotubes as modifiers on the GCE electrode caused a significant change in the electrochemical profile of the surface. This modification expanded the voltammogram area, indicating increased surface activity and enhanced electroactivity induced by the carbon nanotubes. Additionally, two distinct peaks (i.e., anodic and cathodic), were observed, correlating with the redox reactions of metallic iron from the catalyst used in the synthesis of MWCNTs.^39^

**Figure 5:**
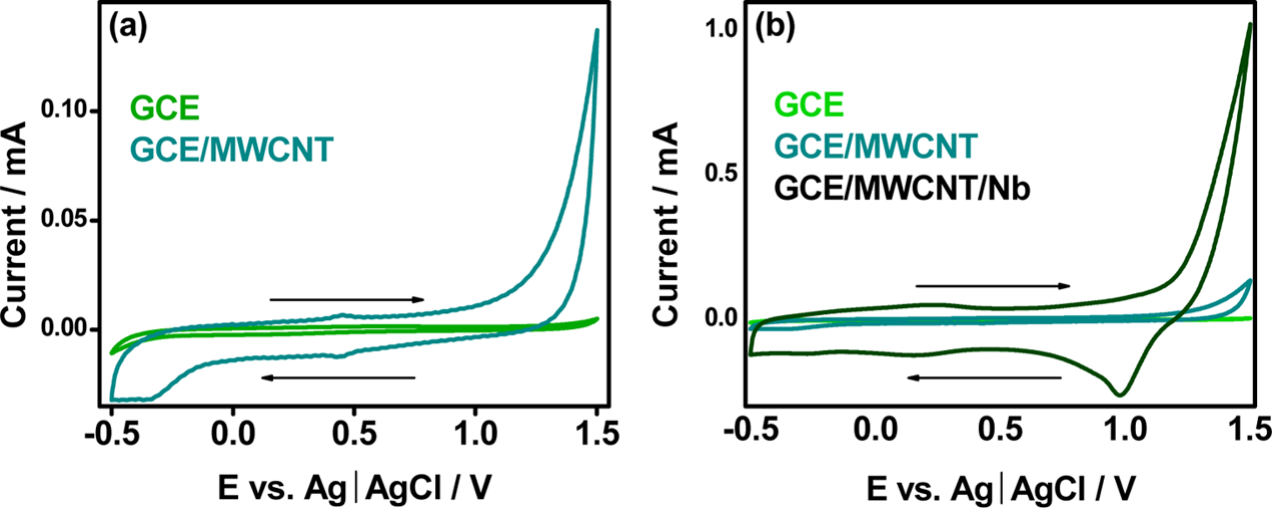
Cyclic voltammograms of the pure GCE electrode and the one modified with MWCNT (a), and a comparative evaluation of the pure GCE electrode, modified with MWCNT, and modified with the nanostructured composites MWCNT/Nb (b), in H_2_SO_4_, 0.50 mol L^–1^, scan rate: 50 mV, step: 0.0244 V.

The voltammetric profile of the modified electrode GCE/MWCNT/Nb, depicted in Figure 5a, stands out for its notable expansion compared to the others. Additionally, the presence of a cathodic peak around 1.0 V, resulting from the reduction of Nb^5+^ to Nb^4+^, is observed.^40,41^ Comparing the voltammetric responses of the modified electrodes with Nb nanoparticles, as illustrated in Figure 5b, clearly demonstrates a superiority in the electrochemical response associated with niobium modification. This indicates that nanostructured Nb enhances the surface area for the adsorption of analytical molecules and increases the electroactive surface compared to other modified electrodes, potentially improving selectivity, sensitivity, and detection limits.

In Figure S3, cyclic voltammograms obtained for the GCE, GCE/MWCNT/Ti, and GCE/MWCNT/Nb electrodes are presented in B–R buffer electrolyte support with pH 7.0. This electrolyte support solution was used for electroanalytical quantifications. The potential range studied was limited to values between −0.20 and 1.0 V, as above 1.0 V, oxygen evolution electrochemical reactions occur, characterized by a rapid increase in current density in this region. Under these new conditions, the GCE/MWCNT/Nb electrode still shows a larger area under the cyclic voltammogram curve than the other electrodes tested. This confirms the superior electroactive response of Nb nanoparticles on MWCNTs compared to Ti-supported MWCNTs and the unmodified GCE surface.

The superior electrochemical response of the GCE/MWCNT/Nb electrode may be attributed to distinctive characteristics of niobium pentoxide, including its range of oxidation states (Nb^5+^, Nb^4+^, and Nb^3+^),^41^ allowing for electron storage and release over a wide range of electrochemical potentials.^42^ In contrast, the redox capacity of titanium dioxide is typically limited to the Ti^4+^ and Ti^3+^ oxidation states, with the latter observed only in specific photocatalytic reactions.^43,44^ Such limitation may result in a lower capacity for detecting electroactive species compared to Nb_2_O_5_. Additionally, the higher current obtained by the GCE/MWCNT/Nb electrode is attributed to the intrinsic electrical conductivity of niobium pentoxide (≈10^–6^ Ω cm^–1^),^45^ which is significantly superior to that of titanium dioxide.^46^ This factor may lead to lower charge transfer resistance of the electrode modified with Nb_2_O_5_, especially when combined with TiO_2_, demonstrating superior electrochemical performance compared to pristine TiO_2_.^47^ The anchoring of Nb_2_O_5_ particles onto carbonaceous materials such as graphene and carbon nanotubes has shown superior synergistic effects compared to the incorporation of TiO_2_ particles into these materials, especially in applications related to supercapacitors and batteries.^48^ This combination results in a significant improvement in redox reaction, increased response current, enhanced chemical stability, and improved capacitive performance of the composite, owing to the contribution of the electronic and ionic conductivity of carbonaceous materials and the high energy storage capacity and electrochemical stability of Nb.

To demonstrate these effects, a preliminary comparative test between the materials was conducted in the detection of hydrogen peroxide (H_2_O_2_) Figure S4. H_2_O_2_ is widely used as a model analyte for the development of electrochemical sensors, and its electrochemical mechanism is well-known.^49^ Cyclic voltammetry assays were conducted to investigate the electrocatalytic activities of GCE/MWCNT/Nb and GCE/MWCNT/Ti electrodes in the presence of H_2_O_2_. The cyclic voltammograms of the electrodes were obtained in Britton–Robinson buffer solution (pH 7.0), with a potential scan from −0.2 to 1.0 V, before and after successive additions of hydrogen peroxide to the cell solution, and the corresponding calibration curves were plotted. The detection limits (LD) and quantification limits (LQ) were calculated according to the IUPAC recommendations as 3 times the standard deviation of the blank signal—B–R buffer—(σ_B_) divided by the slope of the calibration curve (*m*): LD = 3σ_B_/*m*. The LQs were calculated in the same way, with 10 replacing 3 in each equation, i.e., LQ = 10σ_B_/*m*.^50^

Both modified electrodes demonstrated suitability for the electrochemical detection of H_2_O_2_. However, the GCE/MWCNT/Nb electrode exhibited superior sensitivity compared to GCE/MWCNT/Ti. The LD and LQ were 1.5 and 5.0 μmol L^–1^ for GCE/MWCNT/Nb, and 4.0 and 14 μmol L^–1^ for GCE/MWCNT/Ti, respectively. This effect can be attributed to Nb_2_O_5_ nanoparticles containing metal-peroxide species on their surface, resulting from the interaction of Brønsted acid sites (Nb–OH) with H_2_O_2_. This increases surface acidity and, consequently, the adsorption capacity of H_2_O_2_. To validate the reliability of the results, addition and recovery tests were performed in deionized–distilled water for concentration levels of 40 and 60 μmol L^–1^ of H_2_O_2_ in B–R buffer solution pH 7.0 by cyclic voltammetry. The results, presented in Table S1, demonstrated that the GCE/MWCNT/Nb electrode showed excellent recovery capacity, with values ranging between 98% and 106% for the two evaluated concentration levels. On the other hand, the GCE/MWCNT/Ti electrode showed satisfactory recovery values with values ranging between 95% and 101%. Thus, the results indicate that there is no significant difference or superiority between the two sensors, in terms of the recovery capacity of the tested analyte. Peroxide is a model molecule that has been extensively studied in numerous publications. Further analysis of the proposed niobium-based sensor is ongoing, including comparisons with the titanium-based sensor for paracetamol detection in the presence of interferents. These evaluations aim to provide conclusive insights into the performance of the tested sensors and validate the robustness of the MWCNT/Nb sensor.

### 3.3 Evaluation of Square Wave Voltammograms in the Presence of Interference

Square wave voltammetry was employed to determine and quantify paracetamol. The selectivity of the GCE/MWCNT/Ti and GCE/MWCNT/Nb sensors was evaluated for detecting 25 μmol L^–1^ of paracetamol (ACP) in the presence of interferents—25 μmol L^–1^ of epinephrine (EPF) and 50 μmol L^–1^ of tryptophan (TPF)—in Britton–Robinson (B–R) buffer solution (pH 7.0) within a potential range of 0–1.0 V. Figures 6 and S5, show the voltammograms (SWV) for the interferent studies.

**Figure 6:**
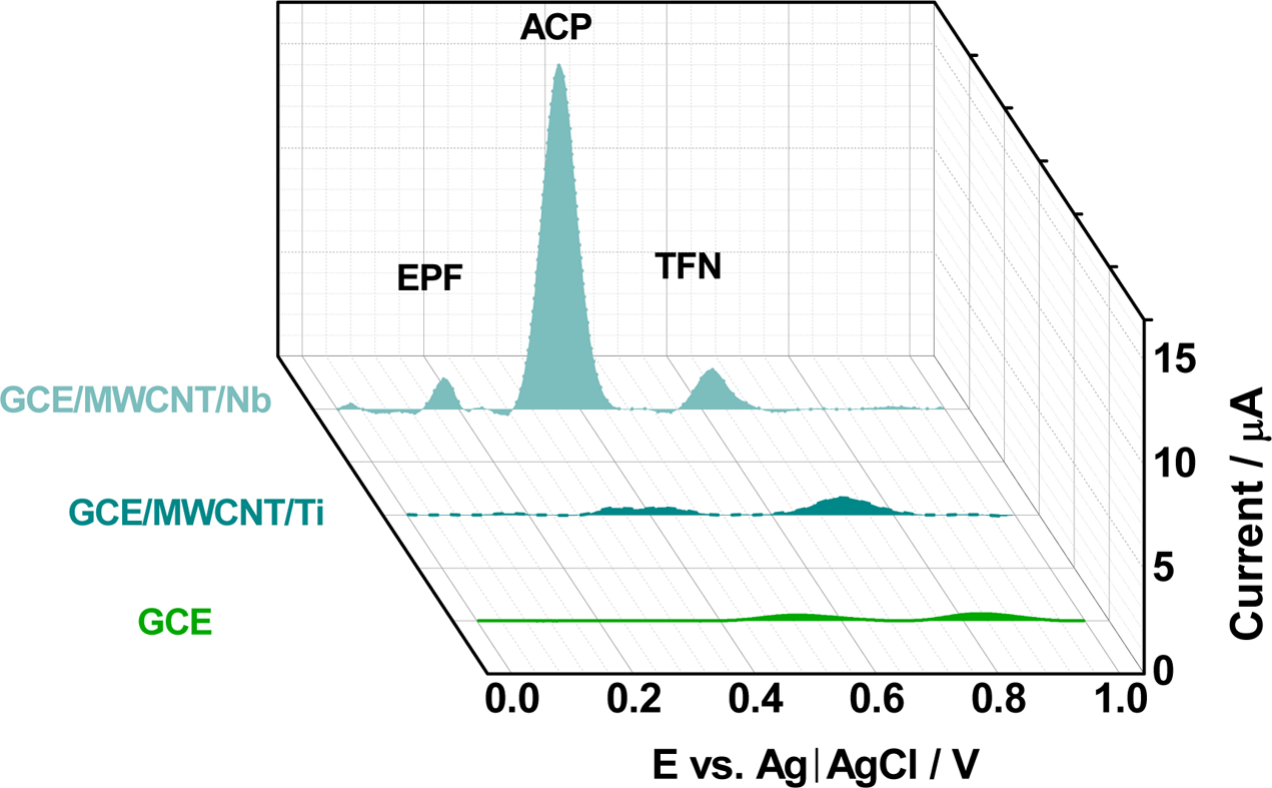
Square wave voltammogram in Britton-Robinson buffer solution (pH = 7) for the working electrodes used in the quantification of 50 μmol L^–1^ of ACP in the presence of interferents containing 25 μmol L^–1^ of EPF and 50 μM of TFN.

Figure 6 highlights the superior performance of the GCE/MWCNT/Nb sensor compared to the bare GCE and the GCE electrode modified with MWCNT/Ti. This superiority can be attributed to stronger chemical interactions between Nb and the species involved in the electrochemical reaction, as Nb exhibits a relatively higher affinity for organic compounds compared to Ti. Consequently, organic compounds are more readily adsorbed onto the surface of Nb, resulting in increased sensitivity and selectivity of the electrode for paracetamol and its interferents. The square wave voltammetry analysis performed with the GCE/MWCNT/Nb electrode yielded remarkable results. The oxidation peaks of ACP (0.4 V) and the interferents EPF (0.2 V) and TFN (0.6 V) were clearly defined, and the GCE/MWCNT/Nb electrode showed higher peak currents compared to the other electrodes studied. The bare GCE electrode (Figure 6) failed to detect epinephrine (0.2 V); only the characteristic oxidation peaks of paracetamol (0.5 V) and tryptophan (0.8 V) could be distinguished. The GCE/MWCNT/Ti electrode, shown in Figure 6, detected only the interferent Tryptophan (0.7 V), with overlapping analytical signals of ACP and EPF. This overlap may be associated with the low affinity for organic compounds exhibited by Ti, making the detection of paracetamol less sensitive and less selective. Given the low selectivity of the GCE/MWCNT/Ti electrode for the target analyte, ACP, under the tested experimental conditions, further studies with this electrode were discontinued.

The analysis of the electrochemical response of the GCE/MWCNT/Nb sensor for detecting 50 μmol L^–1^ of ACP in the presence of interferents (25.0 μmol L^–1^ EPF and 50.0 μmol L^–1^ TFN) over 72 h shows that the sensor maintains consistent performance (Figure S6). During this period, it exhibits a relative response between 90% and 103%, with a relative standard deviation (RSD) of 5.0% compared to the initial measurement taken after electrode preparation. The measurements confirm high electrochemical activity, and the slight decrease observed within the first 48 h indicates that the electrode retains its analytical capability for a significant period. This stability is crucial for practical applications in electrochemical sensors.^51^ The presented data align with reports in the literature, which indicates that CNT-based nanocomposite sensors demonstrate stable performance for at least 48 h of continuous operation, followed by a gradual decline due to electrode passivation or material degradation. Thus, the results confirm that the GCE/MWCNT/Nb sensor remains stable and reliable for up to 48 h, making it suitable for short- and medium-term applications. However, after 72 h, the loss of performance requires electrode replacement or reconditioning to ensure accurate analysis.

### 3.4 Precision and Accuracy of the MWCNT/Nb Sensor

The linear relationship between peak current and paracetamol concentration was meticulously investigated through a calibration curve using an external standard. Various concentrations of paracetamol were added to the B–R buffer solution with a pH of 7.0, ranging from 0.0 to 80.0 μmol L^–1^. The results of this analysis, including the square wave voltammograms obtained and the corresponding analytical curve, are depicted in Figure 7. In Figure 7a, the increase in electrochemical responses of the peaks for both paracetamol and interferents as their concentrations are increased is illustrated. However, the analytical curve in Figure 7b demonstrates a low linear correlation coefficient (*R* = 0.8069), indicating poor linearity. This deficiency may be attributed to the saturation of the electrode surface by interferents, which compromises the linearity of the results.

**Figure 7:**
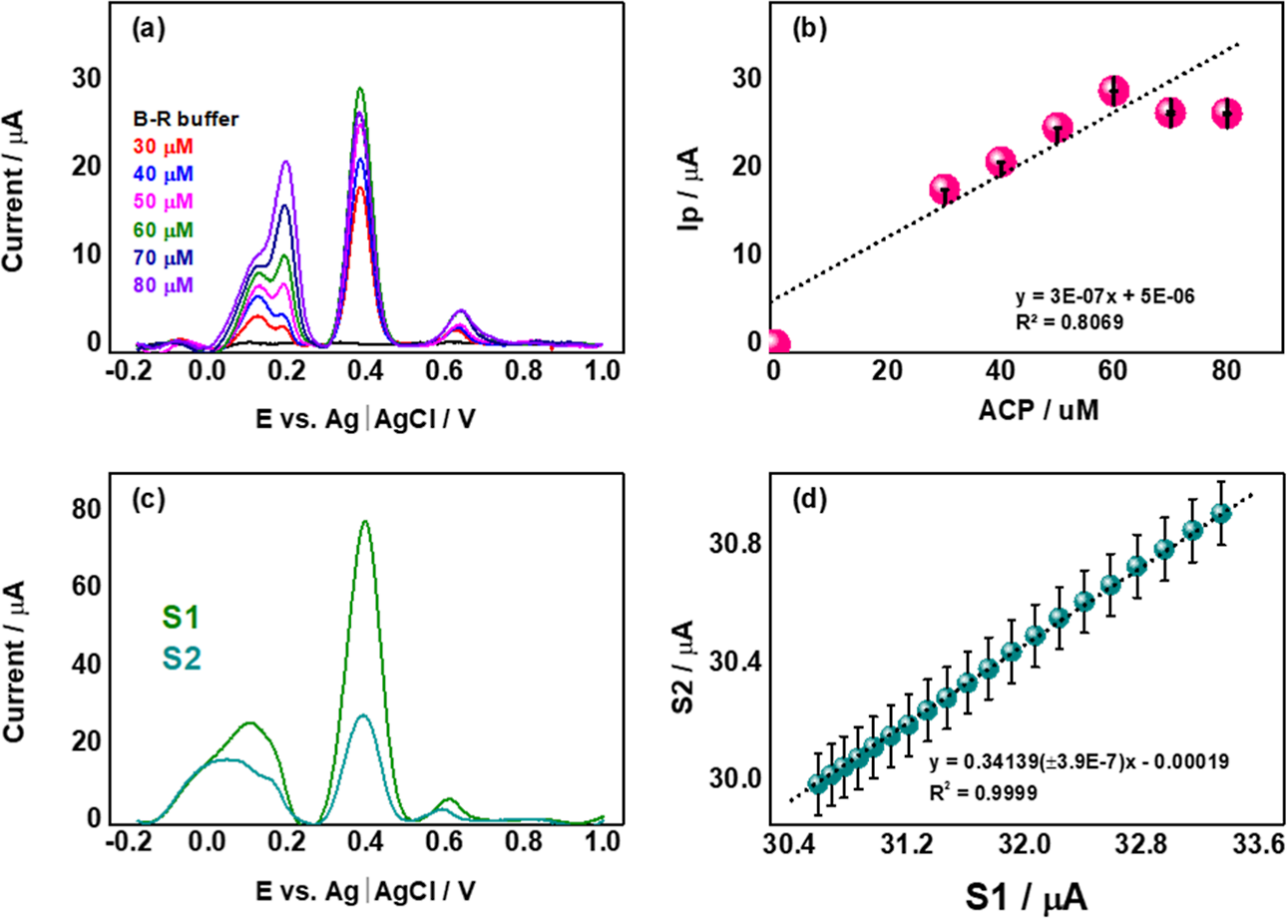
Square wave voltammograms (a) for the paracetamol (0.4 V) at different concentrations in the presence of interferents (epinephrine 0.2 V and tryptophan 0.6 V) in analytes in tap water with B–R buffer solution (pH 7.0). (b) External standard calibration curve. A square wave voltammogram was obtained for calibration curves with all analytes (c) and (d) MEC calibration curves for the analytes in tap water.

Given the limitations in the linear dynamic range of the external calibration method, the MEC methodology was employed to determine the concentration of ACP in water in the presence of interferents.^3^ With MEC, only two calibration solutions are required per sample. Solution S1 comprises 50% of the sample and 50% of a standard solution containing the analytes, while solution S2 consists of 50% of the sample and 50% of a blank (B–R buffer). Figure 7 depicts typical square wave voltammograms (c) and the MEC calibration curve (d) for the three analytes.

The multienergy calibration method enabled the derivation of a calibration curve equation through linear regression, as shown in Figure 7d. The achieved linearity was excellent, with *R* ^2^ = 0.9999. The limits of detection (LD) and quantification (LQ) were calculated following the approach proposed by Virgilio et al.^52^ The LD and LQ values are presented in Table 1 with excellent sensitivity in the order of 10^–9^ molar (nM). Subsequent addition and recovery experiments were conducted to validate the analytical process, as presented in Table 1. Two concentration levels of the sample, Csam (50 μM and 40 μmol L^–1^), and standards, Cstd (100 μmol L^–1^ and 80 μmol L^–1^) were used for this purpose. The results demonstrate the electrode’s exceptional performance, with a recovery percentage of 103%, indicating its efficacy in detecting paracetamol in water in the presence of interferents.

**Table 1:** Results of Addition and Recovery Tests for Determination of Paracetamol in the Presence of Tryptophan and Epinephrine, Carried out Using the MEC Calibration Method at Two Concentration Levels

Cstd (μM)	Csam (μM)	concentration obtained (μM)	recovery (%)	LD (nM)	LQ (nM)
100.0	50.0	51.60 ± 1.83	103.24	0.100	0.500
80.0	40.0	41.20 ± 0.170	103.10		

The detection and quantification limits achieved with the GCE/MWCNT/Nb electrode were compared with those reported in literature for voltammetric detection of paracetamol, as summarized in Table 2. It was found that the electrode modified with MWCNT/Nb is efficient in detecting paracetamol, surpassing the titanium sensor used as a comparative material, with lower detection and quantification limits. Furthermore, this electrode has significant analytical applicability for determining the drug in real samples, as its working range covers the concentrations found in excipients and biological fluids (10–500 μmol L^–1^).^53^

**Table 2:** Comparison of Detection Limit Values Obtained in the Literature for Paracetamol by Different Electrochemical Techniques and Their Surface Modifiers

modifier	electroanalytical technique	LD (μmol L^–1^)	reference
(PVA)-Fe_3_O_4_	DPV	8.00	^54^
TiO_2_	CV	5.20	^55^
PVP/SWCNT	SWV	0.380	^56^
TiO_2_/AuNPs/CMK-3/Nafion	CV	0.210	^57^
Au@Fe-MOF	DPV	0.120	^58^
Po-YPX4R-MCPE	CV	8.40 × 10^–2^	^59^
NiFe_2_O_4_	DPV	8.00 × 10^–2^	^60^
Pd@α-MnO_2_	DPV	5.90 × 10^–2^	^61^
ERGO-SbNPs	DPV	5.70 × 10^–2^	^62^
GO	CV	4.90 × 10^–2^	^63^
TiO_2_-GR	DPV	2.10 × 10^–2^	^64^
BDD	DPV	1.35 × 10^–2^	^65^
AgNPs-CB-PEDOT:PSS	SWV	1.20 × 10^–2^	^66^
N-CeO_2_/rGO	DPV	9.80 × 10^–3^	^67^
MWCNT-ZnO	DPV	3.30 × 10^–3^	^68^
MIP/MoS_2_/CNTs	DPV	3.00 × 10^–3^	^69^
BF/PG	DPV	1.90 × 10^–3^	^70^
CPE/S//NanoCo	DPV	1.00 × 10^–3^	^71^
GrNF/GCE	amperometry	4.00 × 10^–4^	^72^
MWCNTs	SWV	9.90 × 10^–5^	^73^
MWCNT/Nb	SWV	1.00 × 10^–4^	this work

As highlighted in Tables 1 and 2, the MWCNT/Nb sensor exhibits outstanding performance in the electrochemical detection of paracetamol. Its key advantages arise from the synergy between niobium pentoxide and multiwalled carbon nanotubes. This combination enhances sensitivity, surpassing that of the MWCNT/Ti sensor, while also improving selectivity and stability. The broader range of redox states provided by Nb_2_O_5_ facilitates efficient electron transfer, and the high conductivity and large surface area of MWCNTs allow for paracetamol detection at nanomolar levels, even in the presence of interferents like epinephrine and tryptophan. Furthermore, the straightforward synthesis of the MWCNT/Nb sensor ensures reproducibility, making it a promising candidate for analytical applications. While further studies are needed to evaluate its long-term stability and performance in real-world samples, the sensor’s robustness and precision highlight its potential for detecting emerging contaminants in environmental and pharmaceutical settings. By addressing these areas for improvement, the MWCNT/Nb sensor could play a pivotal role in advancing electrochemical detection technologies, providing a reliable and effective solution for complex analytical challenges.

Differential pulse voltammetry—DPV; cyclic voltammetry—CV; square wave voltammetry SWV; poly­(vinyl alcohol)—PVA-Fe_3_O_4_ membranes; titanium dioxide nanoparticle—TiO_2_; single-wall carbon nanotubes (SWCNTs) and polyvinylpyrrolidone (PVP) polymer—PVP/SWCNT/PGE; TiO_2_ enriched with gold nanoparticles (AuNPs) and mesoporous carbon (CMK-3), and stabilized with Nafion—TiO_2_/AuNPs/CMK-3/Nafion; nanocomposite containing gold (Au) nanofibers decorated iron–metal–organic framework (Fe-MOF)—Au@Fe-MOF; Polly yellow (PX4R) modified carbon paste electrode—Po-YPX4R-MCPE; nickel ferrite nanoparticles—NiFe_2_O_4_; palladium atom on the surface of α-MnO_2_ nanorods supported graphene—Pd@α-MnO_2_/G; electrochemically reduced graphene oxide–antimony nanoparticle—ERGO-SbNP; graphene-oxide—GO; TiO_2_–graphene modified—TiO_2_-GR; boron doped diamond – BDD; silver nanoparticles (AgNPs), carbon black (CB), and poly­(3,4-ethylenedioxythiophene)-poly­(styrenesulfonate) (PEDOT:PSS)—AgNPs-CB-PEDOT:PSS; nitrogen doped lanthanum metal oxide with reduced graphene oxide sheets—N-CeO_2_/rGO; multiwalled nanotubes (MWCNTs) and a graphite electrode, to which ZnO nanoparticles—MWCNT-ZnO; hybrid 1T/2H phase molybdenum disulfide (MoS2)/multiwalled carbon nanotubes, deposited on glassy carbon electrode (GCE) to construct molecularly imprinted polymer (MIP) based sensor—MIP/MoS_2_/CNTs; 5,5′-(oxybis­(4,1-phenylene)­bis­(3-(2-hydroxyphenyl)-1-phenylformazan (BF) onto pencil graphite (PG) electrode—BF/PG; Electrodeposition of cobalt nanoparticles (Nano Co) on the surface of carbon paste electrode (CPE) modified with cellulose (C) (CPE/C//NanoCo) and starch (S) (CPE/S//NanoCo) polymers—CPE/S//NanoCo; multiwalled carbon nanotubes—MWCNTs; graphene nanoflakes modified glassy carbon electrode—GrNF/GCE.

The MWCNT/Nb sensor demonstrates exceptional performance in the electrochemical detection of paracetamol, as highlighted in Tables 1 and 2. Its main advantages stem from the synergy between niobium pentoxide and multiwalled carbon nanotubes. This combination enhances sensitivity, surpassing that of the MWCNT/Ti sensor, while also improving selectivity and stability. The broader range of redox states provided by Nb_2_O_5_ facilitates efficient electron transfer, and the high conductivity and large surface area of MWCNTs allow for paracetamol detection at nanomolar levels, even in the presence of interferents like epinephrine and tryptophan. Additionally, the synthesis of the MWCNT/Nb sensor is straightforward, ensuring reproducibility and making it an attractive option for analytical applications. While the sensor demonstrates stability and reliable performance for up to 48 h, with a gradual decline observed after 72 h, further studies are necessary to explore strategies for extending its operational lifespan and assessing its performance in real-world samples. Nevertheless, its robustness and precision reinforce its potential for detecting emerging contaminants in environmental and pharmaceutical settings. By optimizing electrode reconditioning or replacement protocols, the MWCNT/Nb sensor could significantly contribute to advancing electrochemical detection technologies, offering a dependable solution for complex analytical challenges.

## 4 Conclusion

This study introduced a sensor based on metal oxide nanocomposites synthesized from multiwalled carbon nanotubes (MWCNTs) decorated with niobium, deposited on the surface of a glassy carbon electrode (GCE). The nanocomposites were characterized using SEM, EDS, XRD, and TGA to validate their structural and compositional integrity. The electroactive properties of the material for hydrogen peroxide (H_2_O_2_) detection were benchmarked against a titanium-based MON sensor reported in the literature. Both materials exhibited satisfactory electroanalytical performance for the detection of the model molecule (H_2_O_2_) in aqueous media without interferents.

However, the proposed MWCNT/Nb sensor demonstrated superior performance for the voltammetric detection of acetaminophen using square wave voltammetry. It exhibited excellent sensitivity and precision, even in the presence of interfering species such as epinephrine and tryptophan. The detection and quantification limits for acetaminophen reached nanomolar levels (1.0 × 10^–10^ to 5.0 × 10^–10^ mol L^–1^), with recovery rates up to 103%, outperforming other electroanalytical methods reported in the literature.

This work highlights the potential of MON-based sensors in the development of highly sensitive, selective, precise, and accurate electrochemical platforms for detecting contaminants like acetaminophen in water. The synergistic combination of the excellent electrical conductivity of MWCNTs with the catalytic properties of niobium significantly enhanced the electroanalytical performance compared to pure MWCNTs and the unmodified GCE surface.

Future studies could extend the application of this MON-based sensor system to the detection of other environmental contaminants, paving the way for the development of versatile and robust sensor platforms for environmental monitoring.

## Supplementary Material



## Notes

The results, presented in Table S1, demonstrated that the GCE/MWCNT/Nb electrode showed excellent recovery capacity, with values ranging between 98% and 106% for the two evaluated concentration levels. Detailed characterization of the MWCNTs is available in the Figure S1 and S2. In Figure S3, cyclic voltammograms obtained for the GCE, GCE/MWCNT/Ti, and GCE/MWCNT/Nb electrodes are presented in B–R buffer electrolyte support with pH 7.0. Figure S4. H_2_O_2_ is widely used as a model analyte for the development of electrochemical sensors, and its electrochemical mechanism is well-known. Figure S5, show the voltammograms (SWV) for the interferent studies The analysis of the electrochemical response of the GCE/MWCNT/Nb sensor for detecting 50 μmol L^–1^ of ACP in the presence of interferents (25.0 μmol L^–1^ EPF and 50.0 μmol L^–1^ TFN) over 72 h shows that the sensor maintains consistent performance (Figure S6) (PDF)
