# Supplementary material for: Niobium-Decorated Multiwalled Carbon Nanotubes for Voltammetric Detection of Paracetamol
Source: ACS Omega. 2025 May 7;10(19):19410–21. doi: 10.1021/acsomega.4c10722 (PMC12096203; doi:10.1021/acsomega.4c10722)
Supplement: Supplementary file 1 [file ao4c10722_si_001.pdf]

Supporting Information

Niobium-Decorated Multiwalled Carbon Nanotubes for Voltammetric Detection of Paracetamol

Rafael H. de Oliveira †, Monize M. da Silva ‡, Claudio T. de Carvalho ‡, Daniel A. Gonçalves †,¥, Diogo D. dos Reis †**

† Rafael H. de Oliveira – Institute of Physics, Federal University of Mato Grosso do Sul – UFMS, Campo Grande 79070-900, MS, Brazil.

‡ Monize M. da Silva – Faculty of Exact Sciences and Technology, Federal University of Grande Dourados – UFGD, Dourados 79804-970, MS, Brazil.

‡ Claudio T. de Carvalho – Faculty of Exact Sciences and Technology, Federal University of Grande Dourados – UFGD, Dourados 79804-970, MS, Brazil.

†,¥* Daniel A. Gonçalves – Institute of Physics, Federal University of Mato Grosso do Sul – UFMS, Campo Grande 79070-900, MS, Brazil; Institute of Exact Sciences - ICE, Federal University of Amazonas – UFAM, Manaus 69080-900, AM, Brazil.

†* Diogo D. dos Reis – Institute of Physics, Federal University of Mato Grosso do Sul – UFMS, Campo Grande 79070-900, MS, Brazil.

* Corresponding author: Daniel A. Gonçalves, E-mail: daniel.araujogoncalves@gmail.com ; Diogo D. dos Reis, E-mail: diogo.reis@ufms.br

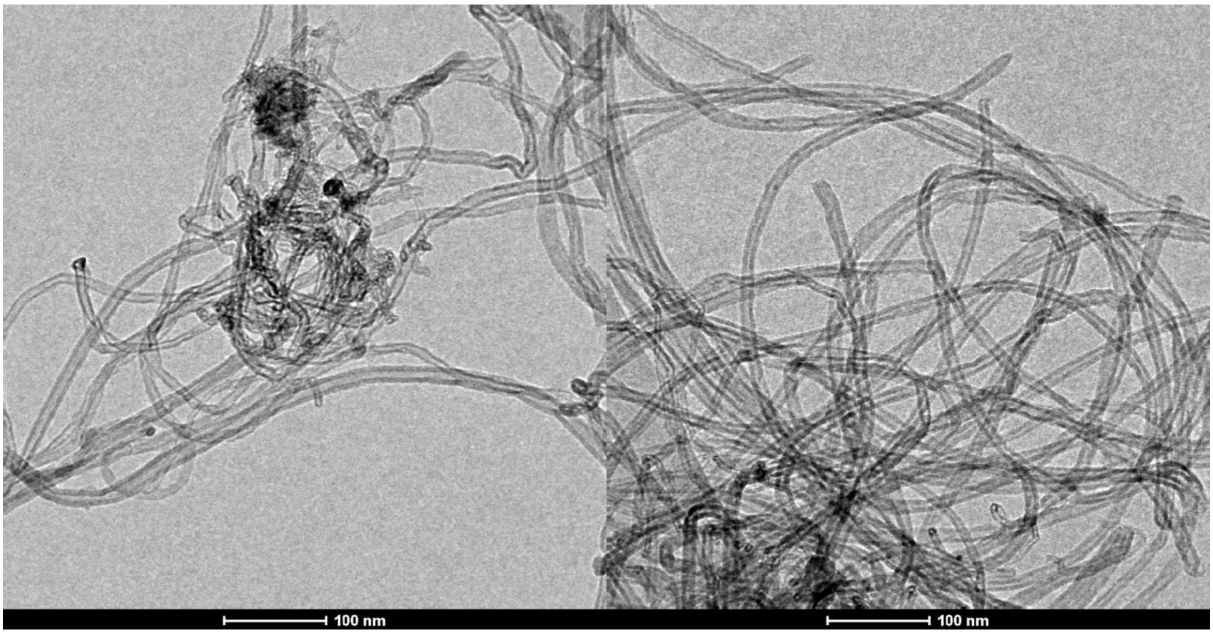

Figure S1. Scanning electron microscopy (SEM) image of multi-walled carbon nanotubes (MWCNTs) synthesized using chemical vapor deposition (CVD), illustrating their surface morphology and structural features.

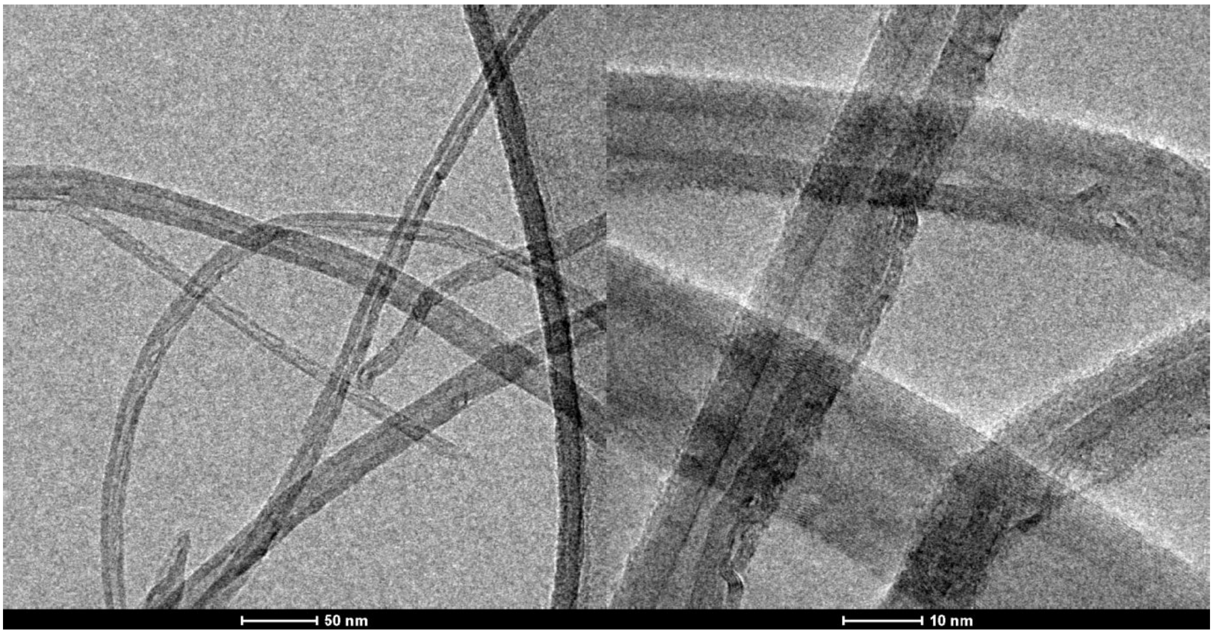

Figure S2. Transmission electron microscopy (TEM) images of multi-walled carbon nanotubes (MWCNTs) synthesized via chemical vapor deposition (CVD), highlighting the internal tube structure and layered wall arrangement.

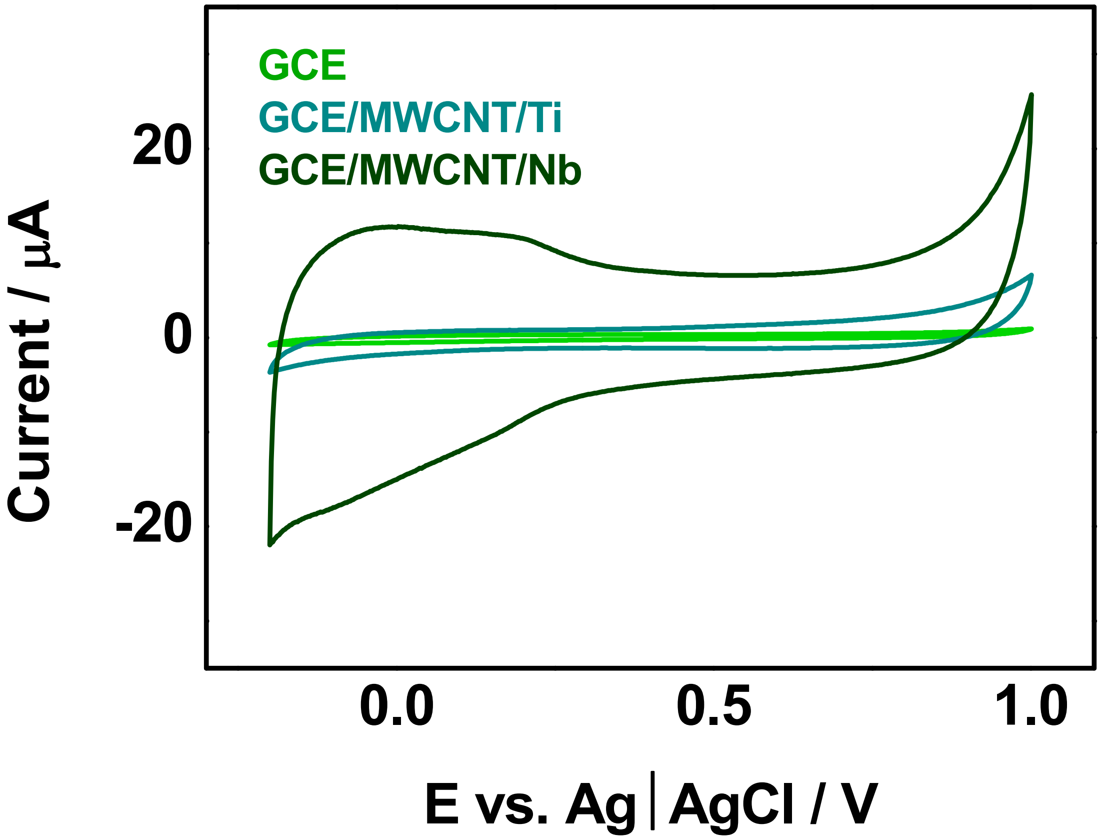

Figure S3. Cyclic voltammograms obtained for the bare glassy carbon electrode (GCE) and modified electrodes with metal oxide nanoparticle-based sensors, specifically GCE/MWCNT/Ti and GCE/MWCNT/Nb. Measurements were conducted in Britton-Robinson (B-R) buffer solution at pH 7.0, with a scan rate of 50 mV/s and a potential increment of 0.0244 V.

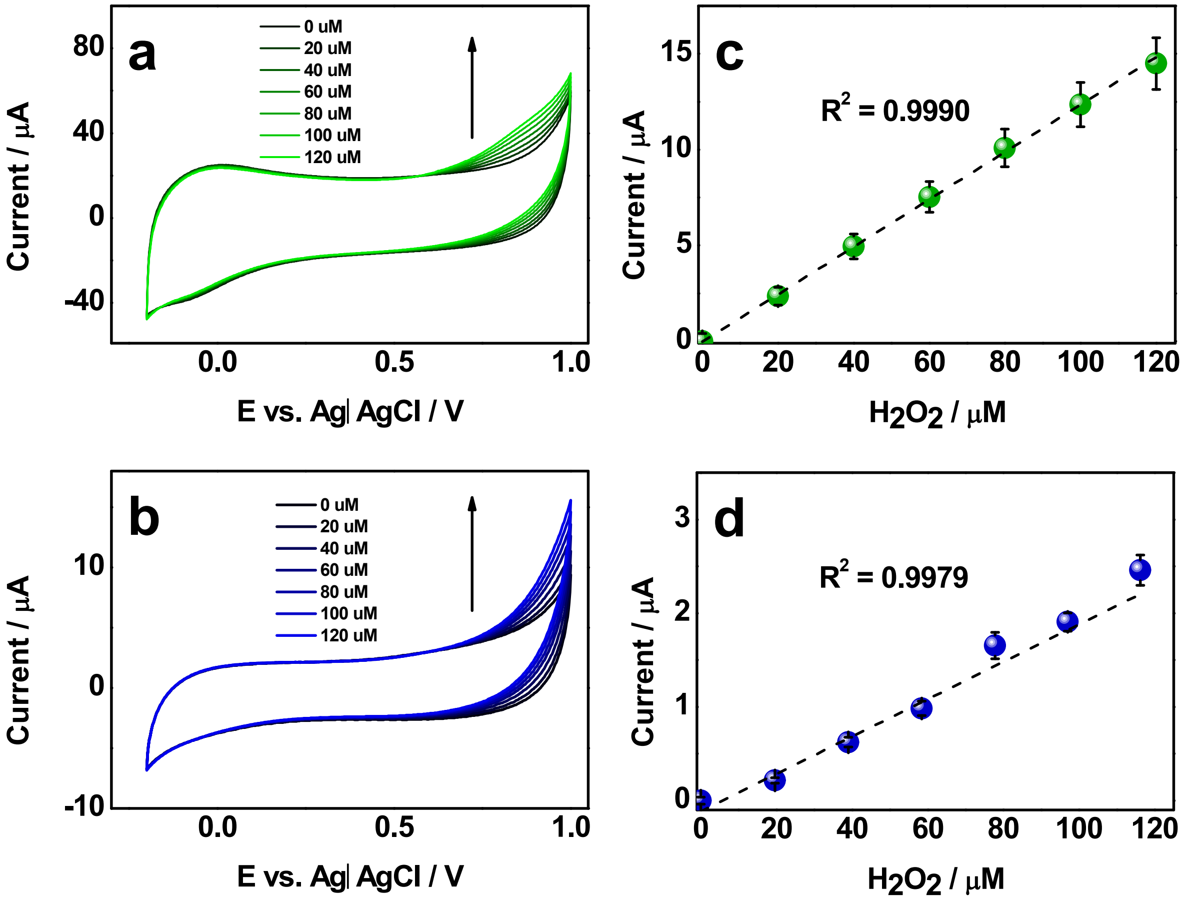

Figure S4. Voltammograms (a - GCE/MWCNT/Nb and b - GCE/MWCNT/Ti) and calibration curves (c - GCE/MWCNT/Nb and d - GCE/MWCNT/Ti) respectively for the MON-based sensors, obtained by cyclic voltammetry for successive additions of H_2O_2 in B-R buffer solution pH = 7.00. Scan speed: 50 mV and step: 0.0244 V. All calibration curves were performed at 0.85 V.

Table S1. Addition and recovery tests in distilled-deionized water, at three concentration levels for H_2O_2 in B-R buffer solution pH = 7.00 by cyclic voltammetry.

MON-based sensors	Concentration prepared (μM)	Concentration obtained (μM)	Recovery (%)
GCE/MWCNT/Nb	40	39.10 ±0.14	98.0
	60	63.33 ±0.25	106.0
GCE/MWCNT/Ti	40	38.04 ±0.20	95.0
	60	60.50 ±0.05	101.0

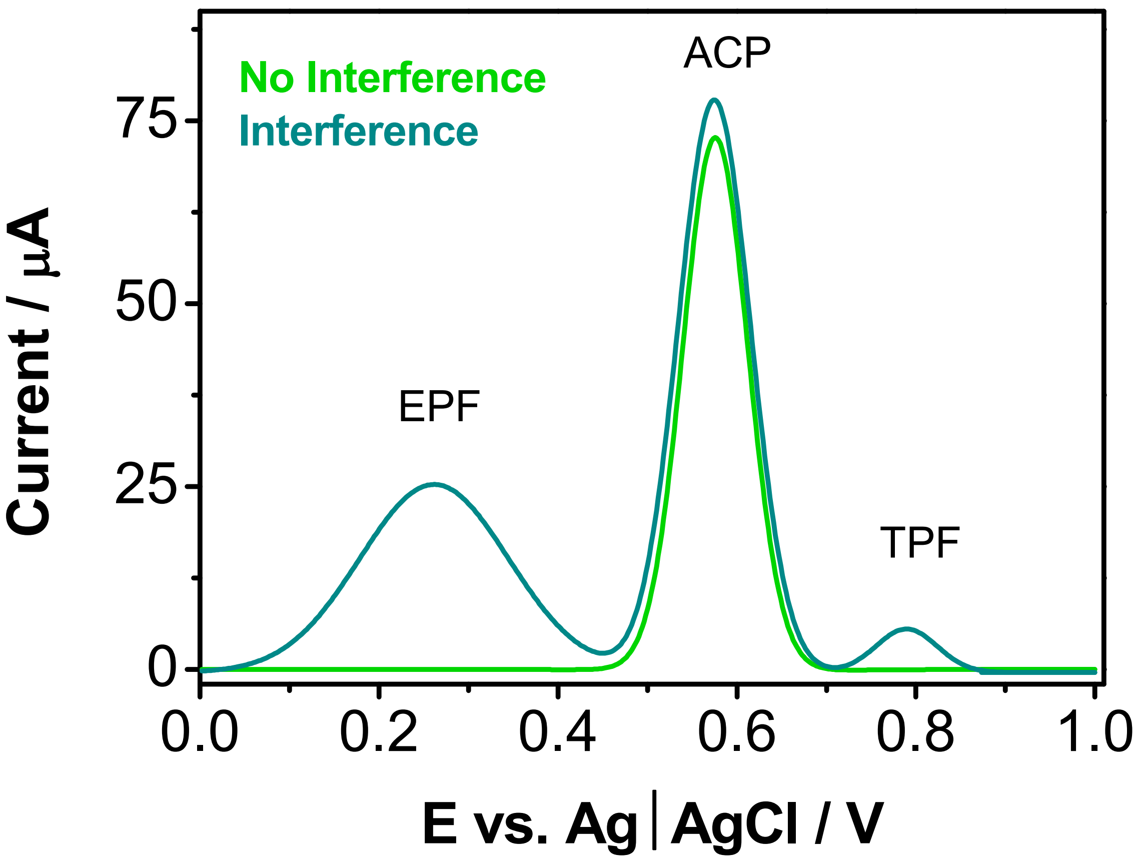

Figure S5. Square wave voltammogram in B-R buffer solution (pH = 7) for the GCE/MWCNT/Nb used in the quantification of 50 µmol/L of ACP in the presence of interferents containing 25 µmol/L of EPF and 50 µM of TFN.

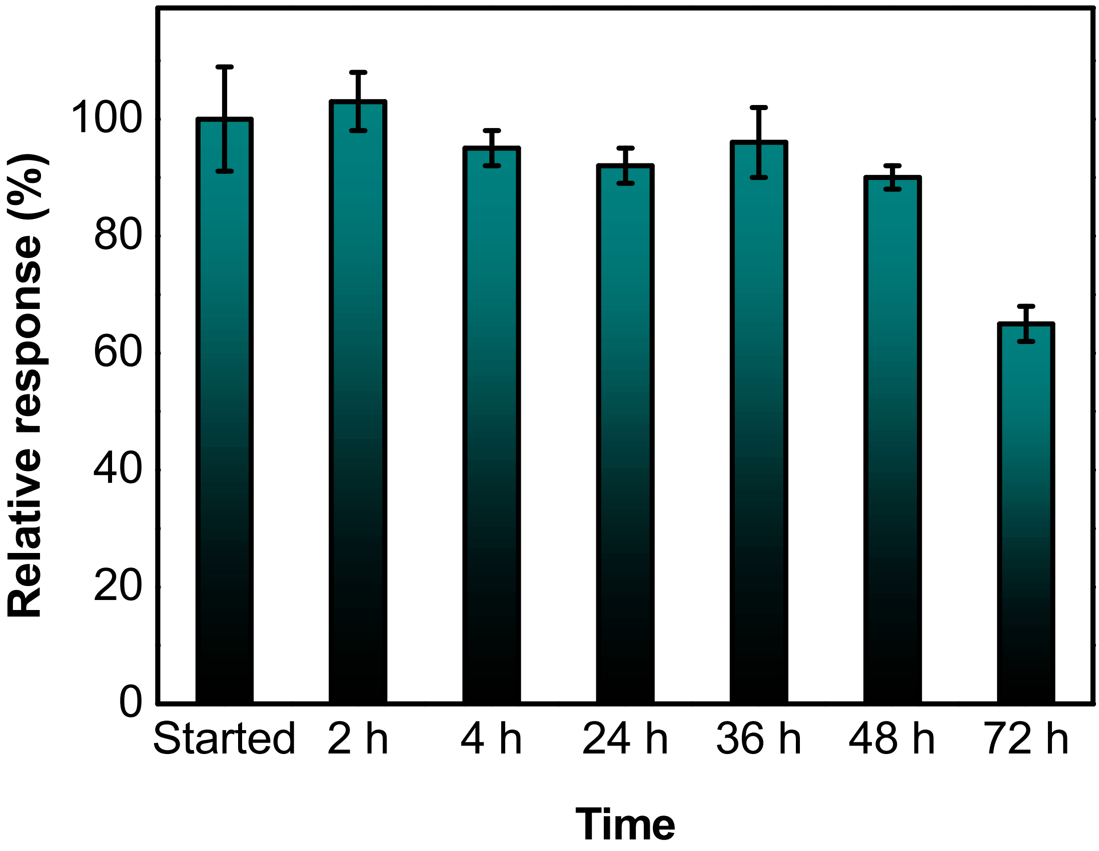

Figure S6. Electrochemical response for the detection of 50 µmol/L of ACP in the presence of interferents (25 µmol/L EPF and 50 µmol/L TFN) using the GCE/MWCNT/Nb sensor over a 72-hour period.
